# Changes in Intakes of Total and Added Sugar and their Contribution to Energy Intake in the U.S.

**DOI:** 10.3390/nu2080834

**Published:** 2010-08-03

**Authors:** Ock K. Chun, Chin E. Chung, Ying Wang, Andrea Padgitt, Won O. Song

**Affiliations:** 1 Department of Nutritional Sciences, University of Connecticut, Storrs, CT 06269, USA; Email: ying.3.wang@uconn.edu; 2 Food and Nutrition, Ansan College, Ansan, Korea; Email: cechung@ansan.ac.kr; 3 Department of Food Science and Human Nutrition, Michigan State University, East Lansing, MI 48824, USA; Email: ajpadgitt@juno.com (A.P.); song@msu.edu (W.O.S.)

**Keywords:** total sugar, added sugar, energy, diet, obesity

## Abstract

This study was designed to document changes in total sugar intake and intake of added sugars, in the context of total energy intake and intake of nutrient categories, between the 1970s and the 1990s, and to identify major food sources contributing to those changes in intake. Data from the NHANES I and III were analyzed to obtain nationally representative information on food consumption for the civilian, non-institutionalized population of the U.S. from 1971 to 1994. In the past three decades, in addition to the increase in mean intakes of total energy, total sugar, added sugars, significant increases in the total intake of carbohydrates and the proportion of carbohydrates to the total energy intake were observed. The contribution of sugars to total carbohydrate intake decreased in both 1–18 y and 19+ y age subgroups, and the contribution of added sugars to the total energy intake did not change. Soft drinks/fluid milk/sugars and cakes, pastries, and pies remained the major food sources for intake of total sugar, total carbohydrates, and total energy during the past three decades. Carbonated soft drinks were the most significant sugar source across the entire three decades. Changes in sugar consumption over the past three decades may be a useful specific area of investigation in examining the effect of dietary patterns on chronic diseases.

## Abbreviations

NHANES: National Health and Nutrition Examination Survey
CVD: cardiovascular diseases
NCHS/CDC: National Center for Health Statistics and the Centers for Disease Control and Prevention
FSD: U.S. Food Supply Data
CSFII: Continuing Survey of Food Intakes by Individuals
HFCS: high-fructose corn syrup

## 1. Introduction

Sugars are a ubiquitous component of our food supply. They are consumed as a naturally occurring component of our diet and as additions to foods during processing, preparation, or at the table. A healthy diet contains at least some amount of naturally occurring sugars, because monosaccharides, such as glucose and fructose, and disaccharides, such as sucrose and lactose, are integral components of fruit, vegetables, dairy products, and many grains [1]. Sugars also add desirable sensory effects and promote enjoyment of foods. Over the years, however, sugar intake has been claimed to be associated with several diet-related chronic diseases: diabetes, CVD, obesity, dental caries, and hyperactivity in children [2,3]. One of overwhelming concerns regarding sugars is the potential for excess energy intake from sugars resulting in weight gain and displacement of more nutrient-dense foods [2]. However, little attention has been given to the contribution of sugar and carbohydrates to total energy intake. 

In explaining the relationship of certain nutrients or dietary patterns to chronic disease, it is important to examine not only the total intake of macronutrients and their components, but also their contribution to total energy intake. We have reported on the association between diabetes biomarkers and increased percent of energy intake from carbohydrates [4], and reported changes in U.S. children’s beverage consumption patterns in the past decades [5]. However, previous studies on the trends of nutrient intakes in the U.S. [6,7,8,9] provided only limited evidence to help explain health impacts associated with consumption of simple and complex carbohydrates, with their ratios to total carbohydrate intake, and with changes in food supply and processing practices. Furthermore, these earlier studies utilized vastly different methodologies, complicating any effort to draw inferences about the relationship of sugar intake to health impacts [10]. 

Trends of sugar consumption in the U.S. have typically been calculated based on the *per capita* sugar consumption estimates reported annually by the Economic Research Service using the market disappearance data [11]. These *per capita* estimates, however, do not take account of differences between the amount purchased and the amount actually consumed. Therefore, it is important to use data on actual consumption, gathered utilizing relatively consistent survey and sampling methods to achieve representative results for the national population. Previous studies analyzing a series of NHANES data documented a steep increase in the prevalence of obesity from mid 1970s through 2000 [12], and a number of studies have shown a significant association between sugar and obesity, especially in children and adolescents [13,14,15]. Therefore, the objectives of this study were to determine trends in the U.S. population and age subgroups, from the 1970s to the 1990s, in total sugar intake and intake of added sugars, in their contribution to total energy intake, and in the food groups contributing principally to sugar and energy intakes. The data for the study were drawn from the National Health and Nutrition Examination Survey, NHANES, I and III (National Center for Health Statistics 1985; 1998). 

## 2. Methods

### 2.1 Characteristics of Datasets

The NHANES I and III were conducted by the National Center for Health Statistics and the Centers for Disease Control and Prevention (NCHS/CDC) through interviews, questionnaires and examinations. The purpose of the surveys was to obtain nationally representative information on the health and nutritional status of the civilian, non-institutionalized population of the U.S. (NHANES I, 1971–1975; NHANES III, 1988–1994) [16,17,18]. Details of survey procedures, handling of samples and analytical procedures are described elsewhere [18]. The characteristics of the NHANES I and NHANES III data sets are shown in Table 1. Data for NHANES I were gathered from 20,195 participants, ages 1 to 74 y, and for NHANES III were gathered from 28,663 participants, ages 1 to 90 y. Subjects with unreliable and incomplete dietary recall records as coded by NCHS were excluded in this study. A listing of the 1,823 unique foods recorded during collection of the 24-hour dietary recall data in the NHANES I was matched to a corresponding food item listed in the NHANES III nutrient database by name and nutrient composition. Both criteria were used to determine the most suitable match for all 1,823 foods. An appropriate match could not be identified for 12 foods (Table 1). 

### 2.2 Study Design

An individual’s total sugar (g·d^−1^) intake was calculated as the sum of glucose, fructose, galactose, sucrose, maltose, and lactose intakes. The definition of “added sugars” was taken from the National Cancer Institute [19]: white sugar, brown sugar, raw sugar, corn syrup, corn syrup solids, high fructose corn syrup, malt syrup, maple syrup, pancake syrup, fructose sweetener, liquid fructose, honey, molasses, anhydrous dextrose, crystal dextrose, saccharin, and aspartame that are eaten separately or used as ingredients in processed or prepared foods. Data of total and added sugars were not available in the NHANES I database. Thus intakes of these nutrients for NHANES I were estimated by matching food codes to those listed in the NHANES III food composition tables. U.S.D.A.’s 53 food categories were used to estimate the food sources of dietary sugar and other nutrients [20]. 

### 2.3 Statistical Methods

All data analyses were carried out using SAS, release 8.1 (SAS Institute Inc, Cary, NC, USA) and Survey Data Analysis for multi-stage sample designs professional software package (SUDAAN, release 8.01, Research Triangle Institute, Research Triangle Park, NC, USA) [21]. SUDAAN was used to increase the validity of the results by computing variance estimates and test statistics for a stratified, multistage probability survey design. Sample weights were applied to all analyses to account for the unequal probability of selection, non-coverage, and non-response bias resulting from over-sampling of low-income persons, adolescents, the elderly, African-Americans, and Hispanics. Means and standard errors for all nutrients examined were calculated using PROC DESCRIPT in SUDAAN. 

**Table 1:** Characteristics of NHANES I and NHANES III datasets.

	NHANES I (1971–1975)	NHANES III (1988–1994)
Sample size	20,195	28,663
Age range	1–74 y	1–90 y
Individual food intake data	353,664	430,050
Number of individual foods	3,462	7,096
in the data set		
Numbers of total sugar is	_	4,000
greater than 0 in NHANES III		
Numbers of added sugar is	_	2,226
greater than 0 in NHANES III		
Number of consumed foods	1,823	4,732
Number of non-matched foods	12^a^	_
Number of matched foods	1,811	_
Nutrient variables in the dataset	energy, protein, fat, carbohydrate, saturated fatty acid,	energy, protein, fat, carbohydrate, saturated fatty acid, oleic acid,
	oleic acid, linoleic acid, cholesterol, vitamin A,	linoleic acid, linolenic acid, monounsaturated fatty acid, polyunsaturated
	thiamin, riboflavin, niacin, vitamin C, calcium,	fatty acid, cholesterol, fiber, alcohol, vitamin A, retinol, β-carotene,
	Phosphorus, iron, sodium, potassium	tocopherol, thiamin, riboflavin, niacin, vitamin B6, vitamin B12, folate,
		vitamin C, calcium, phosphorus, manganese, iron, sodium, potassium,
		glucose, fructose, galactose, sucrose, lactose, maltose, sugar, added sugar
Matched variables with NHANES III	sugar, added sugar, fiber, β-carotene, folate,	_
food composition table	vitamin B6, vitamin B12	

^a ^12 food items in the individual food consumption data file of NHANES I could not be matched, because these items were not described in the food description file of NHANES I.

## 3. Results

### 3.1 Food Code Matches between NHANES I and NHANES III

Estimates of the NHANES I subjects’ nutrient intake levels generated by our food code matching technique (adopted from NHANES III) were comparable to those resulting from analysis of the original food codes of NHANES I. The values resulting from the food code matching technique and the analysis of the NHANES I data were, respectively: for total intake of food and beverages, 2,070 *vs.* 2,070 g·d^−1^; for total energy intake, 1,988 *vs.* 2,000 kcal·d^−1^; for total carbohydrate intake, 224 *vs.* 236 g·d^−1^; and for percent of energy intake from fat, 36% *vs.* 36% (Table 2).

Since the original NHANES I database did not contain sugar intake data, some means of estimating those intakes had to be devised. Since nearly identical values were obtained for the four test nutrient variables from food code matching estimates and from analysis of the original NHANES I data, we felt confident in using the food code matching technique to estimate sugar intake levels for NHANES I participants. 

### 3.2 Changes in Sugar and Added Sugar Intake Levels from NHANES I to NHANES III

Compared with NHANES I, the mean dietary intake levels in NHANES III were greater for total energy intake (+144 kcal d^−1^; +7%), total sugar intake (+10 g d^−1^; +8%), intake of added sugars (+9 g d^−1^; +12%), and total carbohydrate intake (+40 g d^−1^; +18%) (Table 3). The results differed considerably by age subgroup. The change in mean total energy intake for participants ages 1 to 18 was lower by 3%, whereas it was higher by 11% for participants ages 19+ (Table 3). Mean total sugar intake and intake of added sugars increased for participants ages 1–18 by +0% and +5%, respectively, whereas the means for participants ages 19+ increased by +14% and +18%, respectively. 

**Table 2:** Comparison of the mean nutrient intakes of the subjects in the NHANES I estimated based on the original and matched data.^a,b^

Nutrient	Means from original data^c^ (N = 20,195)	Means from matched data^d ^(N = 20,195)	Difference^e ^(%)
Energy (kcal·d^−1^)	1,988	2,000	1
Carbohydrate (g·d^−1^)	224	236	5
Protein (g·d^−1^)	79	76	−4
Fats (g·d^−1^)	82	81	−1
%Energy from fats (%)	36	36	0
Saturated fatty acid (g·d^−1^)	30	31	3
Cholesterol (mg·d^−1^)	372	329	−12
Calcium (mg·d^−1^)	856	858	0
Iron (mg·d^−1^)	12	13	8
Sodium (mg·d^−1^)	2,262	2,901	28
Vitamin A (I.U.·d^−1^)	4,728	4,783	1
Thiamin (mg·d^−1^)	1.1	1.6	45
Riboflavin (mg·d^−1^)	1.8	2.1	17
Niacin (mg·d^−1^)	17	20	18
Vitamin C (mg·d^−1^)	85	90	6
Phosphorus (mg·d^−1^)	1,253	1,225	−2
Potassium (mg·d^−1^)	2,325	2,627	13
Total grams of food or beverage (g·d^−1^)	2,070	2,070	0

^a^ Sample includes those with reliable and complete dietary interview data. ^b^ Means are sample-weighted. ^c^ Nutrient intakes were calculated from original data of NHANES I (1971–1975). ^d^ Nutrient intakes were estimated by NHANES III food composition table through matching food codes of NHANES I to NHANES III. ^e^ Percent differences of matched means compared with original means.

### 3.3 Sources of Energy and Sugars in the U.S. Diets

Appendix A shows the changes in major contributing food items, from NHANES I to NHANES III, for participants ages 1–18 y. Major contributing food items for total energy intake changed (in descending order of importance) from fluid milk/breads/meats to mixtures of mainly grain/fluid milk/breads. Major contributing food items for total carbohydrate intake changed from breads/fluid milk/carbonated soft drinks to carbonated soft drinks/mixtures of grain/breads. Major contributing food items for total sugar intake changed from fluid milk/carbonated soft drinks/cakes, pastries, pies to carbonated soft drinks/ fluid milk/fruitades and drinks. Major contributing food items for intake of added sugars changed from carbonated soft drink/candies, sweets/cakes, pastries, pies to carbonated soft drinks/fruitades and drinks/candies, sweets. 

Appendix B shows the changes in major contributing food items for adult participants (age 19+ y) for the same period. Major contributing food items for total energy intake changed from meats/breads/fluid milk to mixtures of mainly grain/breads/mixed meat dishes. Major contributing food items for total carbohydrate intake changed from breads/carbonated soft drinks/cakes, pastries, pies to breads/carbonated soft drinks/mixtures of grain. Major contributing food items for total sugar intake changed from carbonated soft drinks/fluid milk/sugars to carbonated soft drinks/cakes, pastries, pies/fluid milk. Major contributing food items for intake of added sugars changed from carbonated soft drinks/sugars/cakes, pastries, pies to carbonated soft drinks/cakes, pastries, pies/sugars. 

The most salient feature of the changes in food items contributing to total energy intake is the rise of “mixtures of mainly grain” from relatively insignificant to the most significant contributor in both age subgroups. This food item includes mixtures having a grain product as a main ingredient, such as burritos, tacos, pizza, egg rolls, quiche, spaghetti with sauce, rice and pasta mixtures; frozen meals in which the main course is a grain mixture; noodle and rice soups; and baby-food macaroni and spaghetti mixtures [20].

**Table 3:** Comparison of the mean daily nutrient intakes between the NHANES I (1971-75) and NHANES III (1988-94).^a, b^

	All age			1–18 y			19+ y		
Nutrient	NHANES I	NHANES III	Mean difference^c^ (%)	NHANES I	NHANES III	Mean difference (%)	NHANES I	NHANES III	Mean difference (%)
	n = 20,195	n = 28,663		n = 7,090	n = 12,715		n = 13,105	n = 48,159	
Total sugar (g·d^−1^)^d, e^	120	130	8	138	139	0	110	126	14
Added sugar (g·d^−1^)^e, f^	77	86	12	88	92	5	71	84	18
Calories (kcal·d^−1^)	1,988	2,132	7	2,018	1,962	−3	1,972	2,198	11
Total fats (g·d^−1^)	82	82	0	83	75	−10	81	85	5
Percent energy from total fat (%)	36	34	−6	37	34	−8	36	34	−6
Saturated fatty acid (g·d^−1^)	30	29	−3	32	28	−13	30	29	−3
Cholesterol (g·d^−1^)	372	269	−28	328	225	−32	396	286	−28
Total carbohydrates (g·d^−1^)	224	264	18	244	259	6	213	266	25
Dietary fiber (g·d^−1^)^e^	13	16	19	13	13	0	14	17	25
Protein (g·d^−1^)	79	78	−1	76	68	−11	80	82	2
Calcium (mg·d^−1^)	856	837	−2	1,043	908	−13	755	810	7
Iron (mg·d^−1^)	12	15	29	11	14	33	12	16	26
Total vitamin A (IU·d^−1^)	4,728	5,916	25	4,187	4,565	9	5,021	6,438	28
Beta Carotene (µg·d^−1^)^e^	1,929	2,535	31	1,614	1,694	5	2,100	2,860	36
Folate (µg·d^−1^)^e^	234	265	14	242	238	−1	229	275	20
Vitamin B-6 (mg·d^−1^)^e^	1.6	1.8	13	1.5	1.6	3	1.6	1.9	17
Vitamin B-12 (µg·d^−1^)^e^	5.3	5.3	0	4.9	4.4	−10	5.5	5.6	2
Thiamine (mg·d^−1^)	1.1	1.7	57	1.1	1.7	48	1.1	1.8	61
Riboflavin (mg·d^−1^)	1.8	2.0	14	2.0	2.0	4	1.7	2.0	20
Vitamin C (mg·d^−1^)	85	90	5	83	89	7	87	91	5
Total grams of food or beverage (g·d^−1^)	2,070	2,289	11	1,722	1,718	0	2,259	2,510	11

^a ^Sample includes those with reliable and complete dietary interview data. ^b^ Means are sample-weighted. ^c ^Percent differences of means of NHANESIII compared with NHANES I. ^d^ Total sugar is the sum of total glucose, fructose, galactose, sucrose, lactose, and maltose intakes. ^e^ Intakes of total sugar, added sugar, fiber, beta carotene, folate, vitamin B6, and vitamin B12 in NHANES I were estimated by NHANESIII food composition table through matching food codes of NHANES I to NHANES III. ^f ^One teaspoon of added sugars is converted to the quantity of a sweetener that contains the same amount of carbohydrate as 4.1 g of table sugar.

**Table 4:** Comparisons of food items which contribute to nutrient intake between the NHANES I and III by age subgroups.

Age	1–18 y				19+ y			
Survey	NHANES I		NHANES III		NHANES I		NHANES III	
Energy	Fluid milk	321	Mixtures of mainly grain	230	Meats (beef, pork, lamb, veal)	236	Mixtures of mainly grain	175
(kcal d^−1^)	Yeast breads and rolls	193	Fluid milk	155	Yeast breads and rolls	204	Yeast breads and rolls	170
	Meats (beef, pork, lamb, veal)	150	Yeast breads and rolls	129	Fluid milk	156	Mixtures mainly meat, poultry, fish	151
	Potatoes	105	Regular carbonated soft drinks	105	Cakes, pastries, pies	104	Meat (beef, pork, lamb, veal)	123
	Cakes, pastries, pies	98	Potatoes	100	Fats and oils	95	Cakes, pastries, pies	110
Carbohydrate	Yeast breads and rolls	36	Regular carbonated soft drink	27	Yeast breads and rolls	38	Yeast breads and rolls	31
(g d^−1^)	Fluid milk	26	Mixtures of mainly grain	25	Regular carbonated soft drink	16	Regular carbonated soft drink	27
	Regular carbonated soft drink	18	Yeast breads and rolls	23	Cakes, pastries, pies	15	Mixtures of mainly grain	18
	Cakes, pastries, pies	15	RTE cereals	16	Potatoes	13	Potatoes	15
	Potatoes	14	Fluid milk	14	Fluid milk	13	Cakes, pastries, pies	15
Total sugar	Fluid milk	25	Regular carbonated soft drink	27	Regular carbonated soft drink	17	Regular carbonated soft drink	27
(g d^−1^)	Regular carbonated soft drink	18	Fluid milk	14	Fluid milk	13	Cakes, pastries, pies	9
	Cakes, pastries, pies	13	Regular fruitades and drinks	11	Sugar and sugar substitutes	12	Fluid milk	8
	Regular fruitades and drinks	9	Candies, sweets	9	Cakes, pastries, pies	11	Tea	6
	Candies, sweets	8	Cakes, pastries, pies	8	Citrus juices	6	Regular fruitades and drinks	6
Added sugar	Regular carbonated soft drink	18	Regular carbonated soft drink	27	Regular carbonated soft drink	17	Regular carbonated soft drink	27
(g d^−1^)	Candies, sweets	9	Regular fruitades and drinks	10	Sugar and sugar substitutes	12	Cakes, pastries, pies	7
	Cakes, pastries, pies	9	Candies, sweets	9	Cakes, pastries, pies	8	Sugar and sugar substitutes	6
	Regular fruitades and drinks	9	Syrups, jellies and desserts	6	Syrups, jellies and desserts	5	Regular fruitades and drinks	6
	Sugar and sugar substitutes	6	Cakes, pastries, pies	6	Yeast breads and rolls	4	Candies, sweets	5

The major food groups contributing to total sugar intake and intake of added sugars have remained carbonated soft drinks/fluid milk/sugars, cakes, pastries, and pies. Soft drinks were identified as the most significant source of added sugars, contributing 27 g of sugar intakes daily in NHANES III. The percentage of total sugar intake from soft drinks significantly increased by 49% and 39% for ages 1–18 and 19+, respectively, from NHANES I to NHANES III. In contrast, total sugar intake from milk and milk products dropped by 44% in 1–18 y subjects and 46% in 19+ y subjects, respectively, during the same time period. Sugar intake levels from cookies and breakfast grains remained relatively the same during this time period (Table 4). 

### 3.4 Contribution of Individual Sugars to Total Sugar Intakes

**Figure 1:**
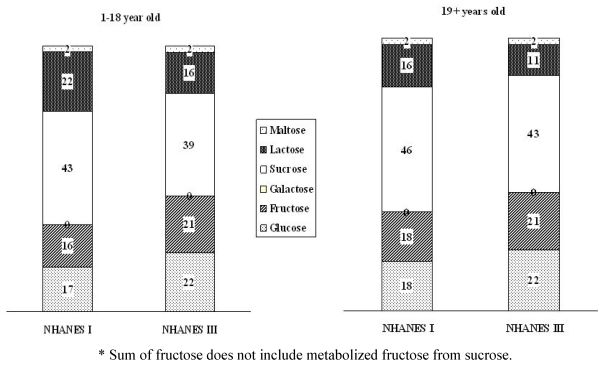
Comparison of the contribution (%) of individual sugars* to the total sugar intakes between the NHANES I and III by age subgroups.

There were differences in the two time periods in the relative contribution of major food groups to average intakes of individual sugars, as a consequence both of changes in food processing and changes in food preferences [22,23]. Carbonated soft drinks, however, remained the greatest contributor to glucose and fructose intakes in all age groups, and fluid milk remained the principal source for lactose intake (Appendix A and Appendix B). Cakes, pastries and pies remained the principal source for sucrose intake in the 1–18 y age subgroup**. **Contribution of glucose and fructose to total sugar intake increased from 17% to 22% (23.4 to 30.7 g·d^−1^) and 16% to 21% (22 to 27 g·d^−1^), respectively, for 1–18 y old subjects and 18% to 22% (20.3 to 27.9 g·d^−1^) and 18% to 21% (21.7 to 29.7 g·d^−1^) for over 19 y old subjects, respectively. Lactose intake has deceased for three decades owing to the decrease in milk consumption and the contribution of lactose to total sugar intake decreased from 22% to 16% (30.9 to 21.6 g·d^−1^) for 1–18 y old subjects and from 16% to 11% (17.3 to 14.2 g·d^−1^) for over 19 y old subjects, respectively (Figure 1). 

## 4. Discussion

Research findings on the assessment of added sugar intake in the U.S. population have been based on two main sources of data: the U.S. Food Supply Data (FSD) series [24] and the Continuing Survey of Food Intakes by Individuals (CSFII) [25,26], both products of the USDA. The Food Supply Series tracks the quantities of foods that flow through the food marketing system. 

The FSD estimates are made at the commodity level. As a result, the data can be used to track changes in the total volumes (and population averages) of specific wholesale products (cane sugar, beet sugar, the various corn sweeteners) that contribute to sugar intake, and categories of their uses (as in beverages and baked goods, for example) [27]. However, since there are losses to domestic use by individual consumers through both waste at various stages of processing, and export, use of these data for population averages requires adjustment of the estimates to account for these losses. The resulting data is therefore less exact than could be hoped for [24]. The CSFII, which has been considered an ideal metric for the concept of added sugars in both Dietary Guidelines and the Food Guide Pyramid [27], provides data on food and nutrient intakes during only 1988–1991, 1994–1996 and 1998. Since 2002, this nationwide dietary intake database has been integrated with NHANES and the data collected as part of NHANES on a yearly basis. The NHANES databases provide a superior longitudinal data source, since they contain earlier data than the CSFII and have a longer period of continuity. 

**Figure 2:**
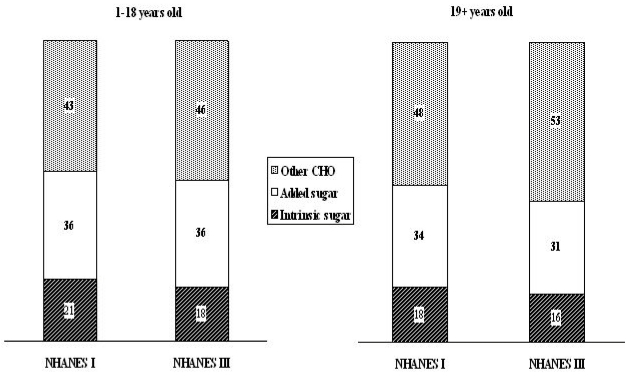
Comparison of the contribution (%) of individual carbohydrates to the total carbohydrate intakes between the NHANES I and III by age subgroups.

The Institute of Medicine [28] reported that people whose diets are high in added sugars have lower intakes of essential nutrients (Ca, Mg, Mg, Fe, Zn, vitamin A and E). It further suggests that added sugars should comprise no more than 25 percent of total calories consumed. In the present study, contribution of each macronutrient to the increased total energy intake was taken into consideration. We observed a significant increase in the total intake of carbohydrates (224 to 264 g·d^−1^) and the ratio of carbohydrates to the total energy intake (45 to 50%), while the contribution of sugars to total carbohydrate intake has decreased in both 1–18 y (57 to 54%) and 19+ y (52 to 47%) (Figure 2); while the contribution of added sugars to the total energy intake has not changed. These findings point to the need for more research into the particular nutritional components related to specific health concerns. 

Several recent studies have suggested total sugar intake and intake of added sugars in the U.S. is related to the development of chronic diseases [26]. Some of these studies in particular identify carbonated soft drinks as a major contributor to energy intake and body weight gain [13,14,15]. Harnack *et al.* [29] reported that children's soft drink consumption had increased during the past three decades by providing 188 kcal·d^−1^ extra energy to soft drink consumers beyond that to non-consumers [29]. St-Onge [15] further suggested that these changes in food intakes among children may partly explain the rise in childhood obesity in the past few years. Adolescents consuming high sugar diets are also reported to be at increased risk for poor health [30] and consumption of sugar-added beverages may contribute to weight gain among adolescents probably due to their contribution to total energy intake [13]. 

The present study shows that energy intake in the 1–18 y subgroup actually decreased during the past three decades, unlike the increase of energy intake among 19+ y age subgroup. In addition, the percentages of energy intake from total carbohydrates increased by 4% and 5% in the 1–18 y and 19+ y age subgroups, respectively, while those from added sugar intake increased by only 1% in both age subgroups. Therefore, even though current trends in health promotion emphasize the importance of increasing carbohydrate intake and reducing fat intake (particularly saturated fat intake), concern has focused on sugar consumption from soft drinks as a main contributor to total energy intake. 

Consumption of added sugars in the U.S. has increased steadily as documented by both FSD and nationwide food consumption survey data. According to U.S. FSD, per capita consumption of added sugars by Americans went from 111 g·d^−1^ in 1970 to 131 g d^−1^ in 1996, an increase of 23% [24]. These data are adjusted for spoilage, other losses accumulated throughout the marketing system and home waste losses. Food consumption survey data also demonstrate an increase in intake of added sugars. According to the USDA CSFII of Americans over 2 y old, consumption of added sugars rose from 64 g·d^−1^ in 1989–1991 to 84 g·d^−1^ in 1994–1996, an increase of 31% in less than ten years. In 1989–1991, added sugars accounted for 13.2% of total daily energy intake, whereas in 1994–1996 they accounted for 15.8% [27]. Although the data from each source indicate an increase in the consumption of added sugars, these increases have not previously been considered in the context of overall changes in macronutrient contribution to total energy intake. Data in the present study confirm the increase in intake of added sugars found in earlier studies, but while the increase in the intake of added sugars during the past three decades was 12% (77 g·d^−1^ to 86 g·d^−1^), its contribution to the energy intake rose less than 4%. This may be too little to account for the increased prevalence in obesity during the same period. Consistently, Sun and Empie [31] failed to find any association between obesity risk and usual sugar-sweetened beverage consumption in adults via analyzing databases of CSFII-1989–1991, CSFII-1994–1998, NHANES III, and combined NHANES 1999–2002 [31]. Animal studies show that carbohydrate-induced obesity is not unique to sweet-tasting sugars, but can also be produced by bland-tasting polysaccharides [32]. These studies as well as the present findings suggest that other carbohydrate categories which contribute more to total energy intake may be more important in examining the growing prevalence of obesity.

A more serious nutritional change related to the increase in intake of added sugars may be the apparent substitution of carbonated soft drink consumption for consumption of fluid milk [5]. Fluid milk was the principal nutritional contributor of energy intake for the 1–18 y age group in the 1970s. Its decreased contribution in the 1990s, and the increased contribution of carbonated soft drinks, may account for much of the decrease in total energy intake and percent energy intake from fat in that age group, as well as the decrease in intakes of calcium and lactose [6]. Overall, the effect of increased intake of added sugars, as it has replaced intake of intrinsic sugars such as lactose and fructose, has been to compromise the intake of more nutritious foods and impeded compliance with current dietary guidelines [6]. 

The amount and type of carbohydrate intake have also received significant attention with increasing prevalence of type 2 diabetes [33], which is highly associated with overweight. The switch from sucrose to high-fructose corn syrup (HFCS) as the sweetener, particularly in the US beverage industry since 1980s, has been suggested to explain the exponential growth of obesity in the U.S. [10] Gross *et al.* [33] reported that increased consumption of HFCS contributed to the increase of energy intakes and consequently to the prevalence of chronic diseases such as type 2 diabetes. Since fructose has higher sweet intensity than sucrose, theoretically the amount of HFCS to yield the same hedonic values would be less than that of sucrose. Clinical and epidemiological studies [10,34,35] have studied the effects of sucrose and fructose on incidence of obesity and other chronic diseases based on the estimates of consumption. Teff *et al.* [10], for example, estimated per capita consumption of added fructose being 81 g·d^−1^. The authors based their estimation of added fructose consumption on the average per capita FSD of 1997 [36], and then combined fructose from HFCS and fructose in the sucrose molecule [10]. In the present study of the NHANES III, we documented that American’s fructose consumption is 30 g·d^−1^ and 27 g·d^−1^ for 1–18 y and 19+ y sub-groups, respectively. Both groups consumed an average of 54 g·d^−1^ of sucrose. 

Differences between the two studies are noteworthy (28 g·d^−1^*vs.* 81 g·d^−1^). We find it important to understand why, in order to assist future investigations in this important area of research. First, per capita disappearance data differ vastly from actual consumption [36]. According to the USDA report [24], loss of refined and beet sugars at retail, food service and consumer levels is estimated to be 31%. Secondly, dietary intake data of an individual or population are reported as consumed in the form of food, beverage and supplements, not in metabolized forms. The USDA [20] and DHHS [17] provide dietary intake data of individual forms of simple sugars, *i.e.*, glucose, fructose, galactose, lactose, sucrose, maltose, *etc.* If one was to estimate the total fructose intake by including fructose metabolized from sucrose, others may argue that glucose metabolized from maltose or starch should be considered in the glucose consumption estimates. Another consideration coming from the study of Duffey and Popkin [37] is that the concept of “total fructose” (including metabolized fructose from sucrose) might hide the truth that fructose consumption has been increasing, because their study showed that total fructose has changed relatively little compared with the change in free fructose and HFCS over the past two decades. American’s per capita consumption of HFCS has increased along with glucose consumption in the U. S. However, the estimated fructose intake cited in the research papers has been overestimated, and might potentially mislead the nutritional science community. 

Our study has limitations. Firstly, since NHANES I included people aged 1–74 years, while NHANES III included people aged 1–90 years, the data for 19+ y subpopulation in the two datasets were not identical. NHANES I (1971–1975) and NHANES III (1988–1994) had different food codes to reflect changes in prevalent dietary behaviors, food commodities and lifestyles in the different time periods. The NHANES I database did not contain estimates of sugar intake levels. Using NHANES I and NHANES III to examine trends in sugar intakes, therefore, required us to develop a food code matching technique. Considering the long time span between the two surveys, the food composition under the same food name might have changed. For example, high-fructose corn syrup (HFCS) has been used as added sweetener, however, the percentage of HFCS of total sweetener has dramatically increased from 0.5% to 37.5%, although total fructose (sum of free fructose and fructose contained in sucrose) availability changed only slightly over the same time period [37]. Although data for HFCS consumption are not available in 1970’s, the results in Table 2 showed that our matching technique was effective and efficient in analyzing unknown sugar information in NHANES I.

## 5. Conclusions

The choice of database is critical in estimating food and nutrient intake. The technique we developed to match food codes in the NHANES datasets allows for their use as a source of reliable data on nutrient and energy intakes in general, and sugar intakes in particular, in the U.S. increased intakes of total and added sugars and carbohydrates have primarily accounted for the increase in energy intakes over the last two decades. The present study indicates that the overall increase in carbohydrate intake has by far exceeded the increase in intake of added sugars, and, thus, more specifically identifies the principal nutritional contribution associated with the rapid rise in obesity in the U.S. over the past three decades. In particular, although soft drink consumption is a major contributor to increased energy intake, the contribution to energy intake from “mixtures of mainly grain” has increased dramatically and is now the principal contributor to energy intake. Increased carbohydrate intake overall is mainly due to the increased availability and consumption of prepared, frozen and takeout meal combinations. Overall, this study points to the need for ongoing research on the specific nutritional contributors to total energy intake, and their potential contribution to increasing prevalence of obesity. 

## Appendix

**Appendix A:** Comparisons of the order of food items which contribute to energy, carbohydrate and sugar intakes of the subjects aged 1–18 y between the NHANES I and III.

Nutrients	Macronutrients								Individual sugars											
Food items	Energy		CHO		Total sugar		Added sugar		Glucose		Fructose		Galactose		Sucrose		Lactose		Maltose	
Survey	I	III	I	III	I	III	I	III	I	III	I	III	I	III	I	III	I	III	I	III
Apples	31	35	19	23	15	18	19	24	8	14	4	5	28	36	14	21	33	47	20	25
Bananas	38	38	28	29	24	22	46	51	14	15	19	15	29	37	18	20	34	42	41	49
Beer	41	40	39	43	42	47	49	52	37	35	30	34	42	48	51	53	45	53	19	16
Cakes, pastries, pies	5	7	4	7	3	5	3	5	11	12	17	21	23	25	1	1	11	12	10	11
Candies, sweets	12	12	7	9	5	4	2	3	4	4	8	10	35	10	3	3	4	7	3	2
Cheese	21	17	43	38	36	35	44	39	47	40	47	41	2	3	47	50	9	6	37	31
Citrus fruits	36	44	25	35	17	28	37	36	13	27	11	20	25	33	15	27	30	44	16	21
Citrus juices	24	23	15	16	8	7	24	22	2	3	2	4	26	34	20	19	31	45	35	48
Coffee	53	53	51	51	52	52	47	45	52	50	52	52	52	40	52	48	52	48	52	50
Cookies	7	13	6	14	9	12	8	8	24	21	21	23	45	51	4	8	13	17	27	17
Crackers	29	27	23	27	34	29	22	21	33	37	32	36	46	21	27	23	49	39	24	35
Creams and cream substitutes	46	45	47	48	40	41	30	31	35	45	43	48	14	28	36	40	16	16	23	27
Dark green vegetables	48	47	46	46	44	43	53	49	34	38	33	35	48	24	40	42	50	19	51	43
Deep yellow vegetables	43	46	36	39	29	36	28	30	31	33	28	33	49	52	26	32	51	31	8	13
Dried fruit	47	49	42	42	35	34	42	44	26	23	23	19	27	35	46	46	32	46	40	39
Eggs	16	24	41	44	30	38	41	43	21	26	48	42	16	13	45	43	12	13	38	32
Fats and oils	8	18	44	41	33	32	15	20	29	30	24	24	32	15	37	33	14	14	42	33
Fish and shellfish	28	31	38	37	48	51	32	33	41	46	39	45	9	26	43	49	48	41	49	53
Fluid milk	1	2	2	5	1	2	34	47	46	51	41	50	7	18	35	51	1	1	28	42
Hot cereals	33	32	27	28	38	30	38	38	28	36	27	30	19	30	34	25	24	15	17	28
Legumes	26	28	20	24	27	37	20	25	27	32	25	31	10	7	23	31	25	29	33	26
Low-calorie carbonated soft drinks	49	52	52	52	53	53	36	34	53	53	53	53	53	43	53	52	53	50	53	51
Low-calorie fruitades and drinks	45	36	34	26	51	13	31	11	50	7	50	6	39	45	49	14	42	51	31	46
Meats (beef, pork, lamb, veal)	3	8	48	47	39	48	51	41	45	47	29	46	1	19	33	41	23	38	47	44
Melons and berries	40	43	31	36	20	26	29	26	20	25	10	18	30	38	16	24	35	33	12	22
Milk desserts	10	16	11	18	6	10	7	7	10	17	22	22	15	5	7	9	2	3	1	3
Milk drinks	18	11	17	10	13	6	12	9	38	19	38	27	13	6	11	6	3	2	22	7
Miscellaneous alcoholic beverages	51	48	53	53	49	50	50	46	51	49	51	49	43	49	39	45	46	35	46	47
Miscellaneous nonalcoholic beverages	42	51	33	49	11	44	10	32	15	44	15	38	40	46	8	36	43	26	45	41
Mixtures of mainly grain^a^	6	1	9	2	21	17	16	16	16	9	14	9	4	1	24	18	10	4	9	9
Mixtures mainly meat, poultry, fish^b^	14	6	22	12	26	20	21	15	23	13	20	12	11	9	28	17	8	8	26	14
Mixtures of mainly vegetables	39	42	35	40	46	42	25	42	43	39	36	37	12	20	44	38	19	25	29	19
Noncitrus juices and nectars	34	25	24	15	18	8	35	23	9	5	7	3	31	14	30	13	37	32	30	30
Nuts and seeds	20	22	32	33	31	33	26	27	32	31	45	29	17	27	25	29	26	37	34	29
Organ meat, sausages, lunchmeat	11	14	40	45	32	39	40	48	25	22	42	44	44	50	32	39	47	36	13	15
Other fruits and mixtures	30	30	18	20	14	16	13	17	7	10	5	7	5	4	13	15	36	24	5	5
Other vegetables	22	29	16	21	23	24	23	29	19	18	16	16	51	12	17	26	20	20	14	23
Pasta	37	37	30	30	41	45	45	50	39	41	35	39	22	32	41	44	28	43	18	18
Popcorn, pretzel, corn chips	27	19	26	19	37	31	27	28	30	28	31	28	24	23	31	30	29	27	15	12
Potatoes	4	5	5	6	25	25	33	40	17	20	13	17	47	8	29	34	7	9	50	40
Poultry	19	10	45	32	45	49	52	53	36	42	37	43	6	22	42	47	22	40	48	52
Quick breads, pancakes, fresh toast	13	15	12	13	22	23	17	18	22	24	26	26	8	17	19	22	5	11	21	20
Regular carbonated soft drinks	9	4	3	1	2	1	1	1	1	1	1	1	37	42	6	2	40	49	6	4
Regular fruitades and drinks	17	20	10	8	4	3	4	2	3	2	3	2	38	44	5	5	41	28	44	36
Rice	32	21	21	11	43	40	39	35	40	34	34	32	21	16	38	35	27	21	39	34
RTE cereals	15	9	8	4	16	9	11	6	18	16	18	14	20	31	10	4	18	23	11	10
Sugar and sugar substitutes	25	39	14	31	7	21	5	12	42	48	44	47	33	39	2	11	38	30	43	38
Syrups, jellies and desserts	23	26	13	17	10	11	6	4	5	6	9	11	34	11	9	10	21	22	2	1
Tea	44	33	37	22	28	15	18	13	49	52	49	51	36	41	22	7	39	34	25	45
Tomatoes	35	34	29	25	19	19	14	14	12	11	12	13	50	53	21	16	15	18	7	6
Wine	50	50	49	50	50	46	48	37	44	43	40	40	41	47	50	37	44	52	32	37
Yeast breads and rolls	2	3	1	3	12	14	9	10	6	8	6	8	18	29	12	12	6	5	4	8
Yogurt	52	41	50	34	47	27	43	19	48	29	46	25	3	2	48	28	17	10	36	24

^a ^Mixtures of mainly grain include mixtures having a grain product as a main ingredient, such as burritos, tacos, pizza, egg rolls, quiche, spaghetti with sauce, rice and pasta mixtures; frozen meals in which the main course is a grain mixture; noodle and rice soups; and baby-food macaroni and spaghetti mixtures. ^b^ Mixtures mainly meat, poultry, fish includes mixtures having meat, poultry, or fish as a main ingredient, such as chicken cacciatore; beef loaf; chili con carne; venison stew; hash; tuna salad; corn dog; chicken soup; frozen meals in which the main course is a meat, poultry, or fish item; meat, poultry, or fish sandwiches coded as a single item (for example, cheese burger on a bun); and baby-food meat and poultry mixtures.

**Appendix B:** Comparisons of the order of food items which contribute to energy, carbohydrate and sugar intakes of the subjects aged 19+ y between the NHANES I and III

Nutrients	Macronutrients								Individual sugars											
Food items	Energy		CHO		Total sugar		Added sugar		Glucose		Fructose		Galactose		Sucrose		Lactose		Maltose	
Survey	I	III	I	III	I	III	I	III	I	III	I	III	I	III	I	III	I	III	I	III
Apples	35	43	22	29	13	19	22	32	10	17	3	4	30	37	18	22	34	44	22	28
Bananas	44	36	29	23	25	18	46	51	19	9	18	14	31	38	22	13	35	42	42	52
Beer	10	12	12	19	28	35	49	52	24	14	15	21	43	50	51	53	46	53	4	3
Cakes, pastries, pies	4	5	3	5	4	2	3	2	11	15	14	18	25	21	2	1	9	12	10	12
Candies, sweets	20	22	15	17	10	8	7	5	12	10	30	23	36	14	4	5	10	11	6	5
Cheese	15	14	42	47	33	37	44	44	46	46	47	44	2	3	47	51	4	5	38	37
Citrus fruits	36	49	23	40	15	29	35	27	13	28	12	22	27	34	13	26	31	46	17	25
Citrus juices	21	28	9	15	5	7	25	47	2	2	2	2	28	35	20	20	32	47	36	50
Coffee	45	41	51	32	52	47	47	28	53	48	52	52	52	42	52	37	52	21	52	32
Cookies	19	18	11	16	12	12	9	10	27	26	23	27	46	52	5	6	16	19	29	20
Crackers	29	31	25	26	38	38	24	23	36	41	34	42	47	25	29	29	49	40	27	34
Creams and cream substitutes	32	37	35	44	30	31	14	20	25	31	43	43	16	30	37	40	5	9	8	11
Dark green vegetables	50	45	46	43	45	44	53	49	35	39	32	33	49	20	39	41	50	17	51	45
Deep yellow vegetables	47	48	34	37	32	30	30	33	32	35	29	30	50	29	24	24	51	37	9	8
Dried fruit	51	51	41	46	36	34	38	45	23	22	21	19	29	36	40	47	33	48	41	44
Eggs	12	19	43	45	31	36	42	41	14	25	48	38	18	12	46	42	12	14	39	30
Fats and oils	5	9	44	38	35	28	15	16	28	24	20	20	13	13	34	30	14	15	30	26
Fish and shellfish	23	23	40	34	49	51	32	34	40	47	36	45	8	22	43	49	48	43	49	51
Fluid milk	3	8	5	10	2	3	36	43	47	51	44	50	1	5	41	46	1	1	33	38
Hot cereals	41	40	31	31	41	46	40	46	33	44	31	41	21	31	36	38	23	18	18	31
Legumes	24	25	21	21	34	32	21	22	30	34	26	31	9	8	25	25	26	25	31	27
Low-calorie carbonated soft drinks	49	53	52	53	53	53	34	29	53	53	53	53	53	45	53	52	53	51	53	53
Low-calorie fruitades and drinks	52	50	47	41	51	26	37	14	50	16	50	12	40	47	49	28	43	35	32	47
Meats (beef, pork, lamb, veal)	1	4	49	50	39	50	51	42	45	50	25	48	3	17	32	44	24	41	47	48
Melons and berries	42	47	28	35	18	23	26	26	16	20	9	15	32	39	14	18	36	36	13	22
Milk desserts	17	17	14	18	7	9	8	7	15	18	22	24	17	6	7	7	2	2	1	2
Milk drinks	33	33	26	27	19	16	13	12	44	32	41	35	15	9	15	10	3	3	24	14
Miscellaneous alcoholic beverages	22	30	53	48	21	33	50	25	51	30	51	25	44	51	11	31	47	28	46	24
Miscellaneous nonalcoholic beverages	48	52	38	52	14	41	10	37	22	37	19	32	41	48	8	34	44	29	45	46
Mixtures of mainly grain^a^	11	1	8	3	26	20	20	19	20	13	17	11	6	2	28	23	13	8	12	10
Mixtures mainly meat, poultry, fish^b^	7	3	19	7	22	15	19	13	18	12	16	10	12	7	27	15	8	4	25	15
Mixtures of mainly vegetables	43	46	36	42	46	48	27	39	39	42	35	39	11	23	38	45	18	22	34	39
Non-citrus juices and nectars	40	44	27	30	20	21	29	31	9	11	8	5	33	40	26	27	38	49	28	35
Nuts and seeds	27	24	37	39	40	39	33	38	37	38	45	36	19	27	31	32	27	39	35	36
Organ meat, sausages, lunchmeat	8	13	45	51	37	43	39	48	26	27	42	47	45	28	33	43	25	34	15	19
Other fruits and mixtures	30	34	18	24	11	13	11	21	6	6	5	6	7	4	12	14	37	32	5	6
Other vegetables	18	20	10	13	16	17	23	24	8	8	10	8	14	11	16	16	20	20	16	21
Pasta	39	35	30	28	48	49	45	50	43	43	38	40	24	33	45	48	29	45	19	23
Popcorn, pretzel, corn chips	38	21	32	22	43	42	31	35	34	36	39	37	26	24	35	35	30	30	21	16
Potatoes	6	6	4	4	24	27	28	36	17	21	13	17	48	16	30	33	11	10	50	40
Poultry	16	10	48	36	47	52	52	53	38	45	33	46	5	19	44	50	22	38	48	49
Quick breads, pancakes, fresh toast	13	11	7	9	23	25	18	18	21	23	28	28	10	18	21	17	6	13	23	18
Regular carbonated soft drinks	9	7	2	2	1	1	1	1	1	1	1	1	38	44	3	4	41	50	7	4
Regular fruitades and drinks	28	27	16	12	6	5	6	4	3	3	4	3	39	46	9	8	42	33	44	42
Rice	31	15	20	6	50	45	41	40	42	40	37	34	23	10	42	39	28	26	40	43
RTE cereals	26	16	17	8	27	14	16	11	29	19	24	16	22	32	19	9	19	23	14	13
Sugar and sugar substitutes	14	29	6	14	3	6	2	3	41	49	40	49	34	41	1	3	39	16	43	33
Syrups, jellies and desserts	25	32	13	20	8	11	4	6	5	5	11	13	35	15	6	11	21	27	2	1
Tea	46	26	33	11	29	4	12	8	49	52	49	51	37	43	17	2	40	31	20	41
Tomatoes	34	38	24	25	17	22	17	17	7	7	7	9	51	53	23	21	17	24	11	9
Wine	37	39	39	49	42	40	48	30	31	33	27	29	42	49	50	36	45	52	26	29
Yeast breads and rolls	2	2	1	1	9	10	5	9	4	4	6	7	20	26	10	12	7	6	3	7
Yogurt	53	42	50	33	44	24	43	15	48	29	46	26	4	1	48	19	15	7	37	17

^a ^Mixtures of mainly grain include mixtures having a grain product as a main ingredient, such as burritos, tacos, pizza, egg rolls, quiche, spaghetti with sauce, rice and pasta mixtures; frozen meals in which the main course is a grain mixture; noodle and rice soups; and baby-food macaroni and spaghetti mixtures. ^b ^Mixtures mainly meat, poultry, fish includes mixtures having meat, poultry, or fish as a main ingredient, such as chicken cacciatore; beef loaf; chili con carne; venison stew; hash; tuna salad; corn dog; chicken soup; frozen meals in which the main course is a meat, poultry, or fish item; meat, poultry, or fish sandwiches coded as a single item (for example, cheese burger on a bun); and baby-food meat and poultry mixtures.
